# Establishing the validity of English GP Patient Survey items evaluating out-of-hours care

**DOI:** 10.1136/bmjqs-2015-004215

**Published:** 2015-10-21

**Authors:** Luke T A Mounce, Heather E Barry, Raffaele Calitri, William E Henley, John Campbell, Martin Roland, Suzanne Richards

**Affiliations:** 1 Collaboration for Academic Primary Care (APEx), University of Exeter Medical School, University of Exeter, Exeter, UK; 2 Department of Public Health and Primary Care, Institute of Public Health, University of Cambridge, Cambridge, UK

**Keywords:** Primary care, Patient satisfaction, Surveys, Quality improvement

## Abstract

**Background:**

A 2014 national audit used the English General Practice Patient Survey (GPPS) to compare service users’ experience of out-of-hours general practitioner (GP) services, yet there is no published evidence on the validity of these GPPS items.

**Objectives:**

Establish the construct and concurrent validity of GPPS items evaluating service users’ experience of GP out-of-hours care.

**Methods:**

Cross-sectional postal survey of service users (n=1396) of six English out-of-hours providers. Participants reported on four GPPS items evaluating out-of-hours care (three items modified following cognitive interviews with service users), and 14 evaluative items from the Out-of-hours Patient Questionnaire (OPQ). Construct validity was assessed through correlations between any reliable (Cochran's α>0.7) scales, as suggested by a principal component analysis of the modified GPPS items, with the ‘entry access’ (four items) and ‘consultation satisfaction’ (10 items) OPQ subscales. Concurrent validity was determined by investigating whether each modified GPPS item was associated with thematically related items from the OPQ using linear regressions.

**Results:**

The modified GPPS item-set formed a single scale (α=0.77), which summarised the two-component structure of the OPQ moderately well; explaining 39.7% of variation in the ‘entry access’ scores (r=0.63) and 44.0% of variation in the ‘consultation satisfaction’ scores (r=0.66), demonstrating acceptable construct validity. Concurrent validity was verified as each modified GPPS item was highly associated with a distinct set of related items from the OPQ.

**Conclusions:**

Minor modifications are required for the English GPPS items evaluating out-of-hours care to improve comprehension by service users. A modified question set was demonstrated to comprise a valid measure of service users’ overall satisfaction with out-of-hours care received. This demonstrates the potential for the use of as few as four items in benchmarking providers and assisting services in identifying, implementing and assessing quality improvement initiatives.

## Introduction

In England, out-of-hours services offer care between 18:30 to 20:00 on weekdays, and on weekends and bank holidays, with care being provided by dedicated general practitioner (GP) services. National audit data reported that these services handled around 5.8 million contacts in 2013–2014, of which, 3.3 million were face-to-face patient consultations.^1^ In England, GP out-of-hours services are contracted to serve local communities by one of 211 Clinical Commissioning Groups;^1^ many providers contract with two or more neighbouring commissioners. Out-of-hours providers are required to report their performance to their commissioners in relation to minimum standards set out in the National Quality Requirements published by the Department of Health.^2^ Recommendation five of the National Quality Requirements mandates providers to regularly audit a random sample of patients’ experiences of the services and to take appropriate action on the results. However, no specific survey tools or methods for auditing patients’ experiences are recommended by the National Quality Requirements, and considerable variation exists in how such audits are conducted. The resultant lack of consensus in methodology impacts on transparency, precluding any attempt to benchmark patients’ views of GP out-of-hours services using data collected to satisfy National Quality Requirement five as direct comparisons between services are not possible. This is problematic as there has been much concern regarding the overall quality and safety of GP out-of-hours care in England and variations in quality.^3^

Patient experience of out-of-hours care is monitored in several healthcare systems. For example, the Patient-Centred Medical Home survey in the USA, which is part of the Consumer Assessment of Healthcare Providers and Systems (CAHPSs) Clinician & Group (CG-CAHPS) survey, contains two questions on out-of-hours care; whether the respondent has been given information on how to obtain care after hours, and whether they are given reminders between visits.^4^ The Australian Bureau of Statistics Patient Experience Questionnaire asks four questions regarding whether respondents have sought out-of-hours care in the previous year and whether they faced barriers to accessing care.^5^ In England, the national General Practice Patient Survey (GPPS) presents respondents with seven items regarding out-of-hours care, including four items for respondents to evaluate the service received (https://gp-patient.co.uk/).

The 2014 English national audit of GP out-of-hours care^1^ presented data on patients’ satisfaction with out-of-hours care provided, taken from the GPPS. Establishing the validity of the GPPS out-of-hours items is an important prerequisite to using this data to document variation in scores between out-of-hours services and then using it to benchmark. We have previously published evidence to support the organisation-level reliability of 45 items used in GPPS, covering six primary care domains (including out-of-hours care), where reliability was estimated with the formula: R=organisation-level variance/(organisation-level variance + residual variance/n)^6^ Of 45 items, 35, including the four items assessing out-of-hours care, were shown to have very good to excellent reliability (R>0.85). Using a range of different methods and analytic approaches, we have also demonstrated the validity of GPPS items evaluating inhours primary care services,^7^ ^8^ but this has yet to be established for evaluative items relating to out-of-hours care.

Our main aim is to ascertain the psychometric suitability of the GPPS items for benchmarking providers and to inform GPPS question design. We explore the validity of four GPPS items evaluating out-of-hours care by comparing their performance with ratings from an established, valid and reliable measure; the Out-of-hours Patient Questionnaire (OPQ).^9^ The OPQ was highlighted as having had rigorous development and good psychometric properties by a recent systematic review of instruments assessing patient satisfaction following teleconsultation and triage.^10^ In addition, the OPQ covers the entirety of service users’ experience of out-of-hours care; from the decision to make contact through to the completion of their care management, allowing assessment of how comprehensive the GPPS items are. The OPQ has been used previously for capturing service users’ experiences of out-of-hours care.^11–13^

Respondents to the GPPS are directed to the out-of-hours questions if they report having contacted an out-of-hours service in the previous 6 months. For this study, we sent postal questionnaires to service users who had contacted one of six English providers in the previous 2 weeks and asked them to report their findings, having considered the last time they contacted an out-of-hours service. It was, therefore, necessary to change the framing of the GPPS items. To ensure the comprehension of our questionnaire, we conducted cognitive interviews^14^ with service users. This process revealed that minor changes to the wording or response options of three of the four GPPS items aided service users’ comprehension. For example, the item ‘How easy was it to contact the out-of-hours GP service by telephone?’ has a response option ‘didn't make contact’, which the cognitive interviewing suggested altering to ‘didn't make contact by telephone’ (further details are provided below). As a result, we reframed the GPPS items to relate to their last contact with an out-of-hours provider and used the modifications to three items advocated by the cognitive interviewing.

We tested the hypothesis that the modified GPPS items will demonstrate construct validity if together they summarise the two known subscales of the OPQ, assessed through correlations. Concurrent validity will be established if thematically relevant OPQ items are found to be associated with each of the GPPS items in linear regressions.

## Methods

### Setting

Six out-of-hours providers across England were recruited for a cross-sectional survey of service users. Providers were sampled using data from Year 5, Quarter 2 (July–September 2010) GPPS (https://gp-patient.co.uk/), with providers selected to ensure there was variation in respect of performance (high/medium/low scoring) on respondents’ overall ratings of care received by GP out-of-hours services, the type of provider (National Health Service (NHS), commercial, social enterprise) and the geographical area covered by the service (inner city/suburban, rural). Two participating service providers were operated by NHS Trusts, three were operated by private (commercial) companies, and one was a social enterprise. Online supplementary table S1 displays the key characteristics of these providers.

### Pilot work

A pilot study was undertaken with two providers. Study questionnaires were distributed to 500 service users (n=250 per provider). Cognitive interviews^14^ were conducted with 20 recent service users (n=10 per provider) to explore their cognitive understanding of the GPPS out-of-hours items in more depth. This preliminary work identified issues with three GPPS questions and sampling of service users. The GPPS filters respondents to the out-of-hours items if they report having tried to make contact with a GP out-of-hours service in the past 6 months, either for themselves or someone else. Since our study respondents were sampled from known service users of out-of-hours providers, we asked respondents to evaluate their experience of the last time they made contact with a GP out-of-hours service. Minor modifications to the wording of the GPPS out-of-hours items (1 item) and/or response options (two items; table 1) and sampling exclusion criteria were proposed by the study team. Prior to commencing data collection, these changes were reviewed and accepted by a study advisory group, which included a service user, three representatives from out-of-hours services, a GP and an external primary care academic.

**Table 1:** Changes made to GPPS items evaluating out-of-hours care following cognitive interviews with service users

GPPS item wording	GPPS response options	Revised wording	Revised response options
Q38. How easy was it to contact the out-of-hours GP service by telephone?	Very easy; fairly easy; not very easy; not at all easy; don't know/didn't make contact	No changes made	Very easy; fairly easy; not very easy; not at all easy; don't know/didn't make contact by telephone
Q39. How do you feel about how quickly you received care from the out-of-hours GP service?	It was about right; it was too long; don't know/doesn't apply	No changes made	It was quicker than expected; it was about right; it was too long; don't know/doesn't apply
Q40. Did you have confidence and trust in the out-of-hours clinician you saw or spoke to?	Yes, definitely; yes, to some extent; no, not at all; don't know/can't say	Did you have confidence and trust in the out-of-hours healthcare professional you consulted with?	No changes made
Q41. Overall, how would you describe your experience of the out-of-hours GP service?	Very good; fairly good; neither good nor poor; fairly poor; very poor	No changes made	No changes made

Changes made to the wording and response options of the four GPPS items evaluating out-of-hours care are underlined.

GPPS, General Practice Patient Survey.

### Description of questionnaire

The questionnaire comprised two sections (see online supplementary appendix). Section 1 included the four modified GPPS evaluative stem items (applicable to all participants). These four items assess service users’ ratings of the ‘entry access’ to the service, the ‘timeliness of care’ received, their ‘confidence and trust’ in the health professional with whom they consulted and their ‘overall experience’ of the out-of-hours service. Section 2 comprised the OPQ, which is composed of seven sections that seek to capture information on the entirety of service users’ experience of out-of-hours care. The composition of the OPQ has been described in detail elsewhere,^9^ where it was found to be both valid and reliable. We analysed the participants’ ratings on 14 evaluative items (table 2) that were not management-specific and that assessed the service users’ experience of entry into the service, the outcome of their call and the consultation with a health professional.

**Table 2:** The Out-of-hours Patient Questionnaire: 14 items used in analyses

Questionnaire section	Item	Response scale
Making contact with the service	How do you rate (how long it took your call to be answered, excluding any introductory message)?	5-point: ‘very poor’ to ‘excellent’
	Please rate the helpfulness of the call operator.	5-point: ‘very poor’ to ‘excellent’
	Please rate the extent to which you felt the call operator listened to you.	5-point: ‘very poor’ to ‘excellent’
	How do you rate (how long it took for a health professional to call you back)?	5-point: ‘very poor’ to ‘excellent’
The outcome of your call	Were you happy with the type of care you received?	Yes/no
The consultation with the health professional	How do you rate (the length of your consultation with the health professional)?	5-point: ‘very poor’ to ‘excellent’
	(Please rate) the thoroughness of the consultation.	5-point: ‘very poor’ to ‘excellent’, plus N/A
	(Please rate) the accuracy of the diagnosis.	5-point: ‘very poor’ to ‘excellent’, plus N/A
	(Please rate) the treatment you were given.	5-point: ‘very poor’ to ‘excellent’, plus N/A
	(Please rate) the advice and information you were given.	5-point: ‘very poor’ to ‘excellent’, plus N/A
	(Please rate) the warmth of the health professional's manner.	5-point: ‘very poor’ to ‘excellent’, plus N/A
	(Please rate) the extent to which you felt listened to.	5-point: ‘very poor’ to ‘excellent’, plus N/A
	(Please rate) the extent to which you felt things were explained to you.	5-point: ‘very poor’ to ‘excellent’, plus N/A
	(Please rate) the respect you were shown.	5-point: ‘very poor’ to ‘excellent’, plus N/A

NA, not applicable.

### Sampling

Sampling took place within 2 weeks of the person contacting the out-of-hours service. The demographic and contact details for a random sample of 2000 service users were extracted from electronic records at each site. Providers then excluded service users if they: were aged 12–17 years, due to the risk of breaching patient confidentiality on account of a questionnaire being sent to the patient's home address, and because the GPPS targets those aged 18+; were admitted to hospital as a result of the contact; had palliative care needs; or if they had a temporary/incomplete address. After all exclusions were applied, a sampling frame of the first consecutive eligible 850 service users (or parent or guardian if the service user was a child) was provided to the research team. A questionnaire, accompanied by covering letters from the service provider and research team, an information sheet and prepaid envelope, was sent to the service users identified at each site. In one area, only 818 service users were sampled, as the sampling frame provided contained a number of duplicate entries, which were excluded. Time constraints prohibited further sampling in this area. Thus, the total sample approached was 5068 service users. A reminder was sent 2 weeks after the initial mailing to non-respondents. Implicit consent was assumed if a completed questionnaire was received by the research team; service users who returned a blank questionnaire were not sent a reminder. Data collection occurred between September 2013 and July 2014.

### Data analysis

Respondents and non-respondents were compared in respect of their age, gender, local area deprivation and management option received as a result of the last recorded contact (from service provider record: telephone advice, treatment centre attendance, home visit) using a multilevel logistic regression model in which ‘clusters’ of patient were identified depending on the provider from which they were sampled. Quintiles of service users’ Index of Multiple Deprivation (IMD) 2010^15^ scores, obtained based on their street address (postcode), were used to determine local area deprivation. The IMD, developed by the English Department of Communities and Local Government, is a composite score of seven domains of deprivation (income, employment, health and disability, education skills and training, barriers to housing and services, living environment and crime).

#### Construct validity

We assessed the construct validity of the four modified GPPS items by determining how well they summarised the OPQ. First, we conducted a confirmatory factor analysis to establish whether the OPQ possessed the same two-factor structure reported in the paper detailing its development: The first proposed subscale consists of four items assessing ‘entry access’ (the ‘making contact with the service’ section; table 2) and the second consists of 10 items assessing ‘consultation satisfaction’ (the ‘outcome of your call’ and ‘consultation with the health professional’ sections; table 2).^9^ We report the standardised factor loadings with 95% CIs for this model. As suggested by Hu and Bentler,^16^ goodness of fit of the model was assessed through a two-index strategy using the Standardised Root Mean Squared Residual (SRMSR) supplemented with the Comparative Fit Index (CFI),^17^ neither of which is adversely affected by large sample sizes.^18^

We then conducted a principal component analysis (PCA) of the four modified GPPS items to establish their latent structure. This PCA used a matrix of polychoric correlations, which are analogous to Pearson's correlations between ordinal variables, as the ranges of items’ response options (table 1) were too restricted to be reasonably considered continuous.^19^ Inspection of eigenvalues and eigenvectors (component loadings) were used to explore the underlying structure of responses. Based on this PCA, we explored the construction of scales using the modified GPPS items and report their internal consistency (Cronbach's α). Finally, we investigated the correlations between the scales constructed above and the factor scores from the confirmatory factor analysis of the OPQ to assess the extent to which the modified GPPS item-set summarised the OPQ.

#### Consultation satisfaction scale

The OPQ contains nine items rating service users’ satisfaction from their consultation with an out-of-hours clinician (table 2). The original study validating the OPQ reports that these items form a ‘consultation satisfaction’ scale with a Cronbach's α of 0.96. In order to avoid over-fitting our regression models and introducing possible issues with multicolinearity, we opted to combine these items into a scale. To achieve this, we linearised each item to a 0–100 scale and then derived respondents’ mean scores from the nine items as their ‘consultation satisfaction’ scale score, provided they had answered at least four of the items. This method has been used previously to derive an inhours GP communication scale using seven GPPS items.^20^ Finally, we standardised the scale so that the regression coefficients obtained from modelling would reflect the change in the dependent variable (each modified GPPS item) produced by an increase in the scale by 1 SD (ie, a standardised coefficient).

#### Concurrent validity

To investigate the concurrent validity of the modified GPPS items, we constructed four mixed-effects, multilevel linear regression models, creating a separate model for each evaluative outcome; ‘entry access’, ‘timeliness of care’, ‘confidence and trust’ and ‘overall experience’. The covariates were the management non-specific items from the OPQ (table 2), including the ‘consultation satisfaction scale’. Concurrent validity was considered to be demonstrated if each modified GPPS outcome was found to be significantly associated with thematically related items from the OPQ (convergent and divergent associations). A literature review was conducted to determine which, if any, OPQ items are thematically related to each of the four outcomes, which is summarised in the discussion. This review revealed little work investigating factors associated with service users’ evaluations of ease of entry access. In this instance, assessment of face validity was used in conjunction with the existing evidence.

Univariate analyses were undertaken first, with covariates being excluded from the final models if they were not associated with any of the four outcomes, as indicated by the *t* statistic for that covariate having a p value greater than 0.1 in the regression model. All models controlled for service users’ age, gender, deprivation quintile (from IMD scores) and management option, as well as the type of provider contacted (NHS, commercial, social enterprise) were clustered by provider. Multiple imputations were used to account for missing data. To ensure that the regression coefficients of covariates were comparable across the models, we standardised the four modified GPPS outcomes, which originally had differing response scales (table 1). Sensitivity analyses were performed to test for a linear trend over the covariate rating length of time taken for a health professional to call back, which modelled the data while excluding those who were ‘not applicable’ (n=192).

All statistical analyses were performed using Stata/SE V.13.

## Results

### Response rate and sample

Completed questionnaires returned within 100 days were received from 1396/5068 (27.6%) of sampled service users. The multilevel logistic regression, assessing response while clustering by provider revealed that responders were older and more affluent (lower IMD score), but did not differ with respect to gender. Differences in response rates were also evident across the management options. Table 3 displays the demographic characteristics and management of responders and non-responders. The item response distributions for all variables of interest are displayed in online supplementary table S2.

**Table 3:** Characteristics of responders and non-responders (n=5067)

	Responders	Non-responders	p Value*
Frequency (%)	1396 (27.6)	3672 (72.4)	
Age in years, mean (SD)	46.0 (28.2)	32.5 (26.2)	<0.001
Gender, female (%)	877 (62.8)	2208 (71.6)	0.081
IMD score, mean (SD)	19.0 (14.0)	23.9 (15.9)	<0.001
Management			
Telephone advice (%)	492 (35.2)	1143 (38.5)	0.001
Treatment centre (%)	647 (46.4)	1765 (48.1)	
Home visit (%)	172 (12.3)	301 (8.2)	
Other (%)	85 (6.1)	193 (5.3)	

*Reported p values were obtained from a multilevel logistic regression that compared responders with non-responders. The model clustered individuals by the provider from which they were sampled.

IMD, Index of Multiple Deprivation.

### Construct validity

#### Confirmatory factor analysis of the OPQ

The confirmatory factor analysis revealed that the data fit the proposed ‘entry access’ and ‘consultation satisfaction’ two-factor structure moderately well (table 4), with a SRMSR of 0.06 (values under 0.08 represent good fit)^16^ and a CFI of 0.89, which is just short of the suggested cut-off of 0.90 for good fit.^16^ The two latent variables were moderately correlated (r=0.54, p<0.001).

**Table 4:** Confirmatory factor analysis of the Out-of-hours Patient Questionnaire

	Coefficient*	95% CI	p Value
*Entry access*			
How do you rate (how long it took your call to be answered)?	0.65	0.61 to 0.70	<0.001
Please rate the helpfulness of the call operator.	0.91	0.89 to 0.93	<0.001
Please rate the extent to which you felt the call operator listened to you.	0.90	0.88 to 0.92	<0.001
How do you rate (how long it took for a health professional to call you back)?	0.66	0.62 to 0.70	<0.001
*Consultation satisfaction*			
Were you happy with the type of care you received? (no/yes)	0.47	0.41 to 0.52	<0.001
How do you rate (the length of your consultation with the health professional)?	0.80	0.77 to 0.83	<0.001
(Please rate) the thoroughness of the consultation.	0.88	0.86 to 0.89	<0.001
(Please rate) the accuracy of the diagnosis.	0.84	0.81 to 0.86	<0.001
(Please rate) the treatment you were given.	0.86	0.84 to 0.88	<0.001
(Please rate) the advice and information you were given.	0.90	0.88 to 0.91	<0.001
(Please rate) the warmth of the health professional's manner.	0.87	0.85 to 0.89	<0.001
(Please rate) the extent to which you felt listened to.	0.93	0.92 to 0.94	<0.001
(Please rate) the extent to which you felt things were explained to you.	0.92	0.91 to 0.93	<0.001
(Please rate) the respect you were shown.	0.86	0.84 to 0.88	<0.001

*The coefficients in a standardised confirmatory factor analysis can be interpreted as correlation coefficients to the latent variable (or factor loadings).

#### PCA of the modified GPPS items

The PCA of the polychoric correlation matrix of the four modified GPPS items extracted a single component with an eigenvalue exceeding 1.0 (2.78), which accounted for 69.5% of the variance in the data. Observed eigenvectors were 0.44 for ‘entry access’, 0.47 for ‘timeliness of care’, 0.51 for ‘confidence and trust’ and 0.57 for ‘overall experience’. This component can be interpreted as overall satisfaction with out-of-hours care. A rotation was unnecessary, as simple structure was obtained.

Informed by the PCA, we investigated the construction of an ‘overall satisfaction’ scale using all four items. This scale was created by summing the standardised items (to account for differing response scales), if responses were given to all items. The ‘overall satisfaction’ scale had acceptable internal consistency, α=0.772. Excluding the ‘entry access’ item suggested a very minor improvement in α, α=0.777 (see online supplementary table S3).

#### How well do the modified GPPS items summarise the OPQ?

The ‘overall satisfaction’ scale was reasonably well correlated with the factor scores of both OPQ domains for ‘entry access’ (r=0.63, p<0.001, r^2^=0.397) and ‘consultation satisfaction’ (r=0.66, p<0.001, r^2^=0.440). Both these correlations are greater than the correlation reported between the two OPQ domains. Thus, when combined into a single scale, the four modified GPPS items explain 39.7% of the variation in ‘entry access’ factor scores and 44.0% of the variation in ‘consultation satisfaction’ factor scores, summarising both scales moderately well. An inspection of table 4 reveals that the ‘entry access’ domain of the OPQ was most related to service users’ experience of the call operator, for which, there is no equivalent GPPS item, perhaps explaining the lower correlation between the ‘overall satisfaction’ scale and the ‘entry access’ factor scores.

### Concurrent validity

Multiple imputation of missing data (see online supplementary table S2) allowed for incorporation of all 1396 respondents in the four mixed-effects, multilevel linear regressions. A distinct pattern of associations across the covariates was evident between the models for each of the four GPPS outcomes (table 5). The item assessing ‘entry access’ was strongly associated with service users’ evaluations of the time taken to answer their call, the helpfulness of the call operator and the time taken for a clinician to call them back (where applicable). The ‘timeliness of care’ item was more strongly associated with the time taken for a clinician to call them back than any other GPPS item, and furthermore was related to the time taken for their call to be answered. ‘Confidence and trust’ was more strongly associated with consultation satisfaction than any of the other modified GPPS outcomes. Finally, ‘overall experience’ was strongly associated with the helpfulness of the call operator, the time taken for a clinician to call them back and consultation satisfaction.

**Table 5:** Linear regression models showing the associations of OPQ items to the four modified GPPS outcomes

Covariate	Entry access		Timeliness of care		Confidence and trust		Overall experience	
	Coefficient (95% CI)	p Value	Coefficient (95% CI)	p Value	Coefficient (95% CI)	p Value	Coefficient (95% CI)	p Value
Call answer time	0.13 (0.06 to 0.21)	0.001	0.09 (0.03 to 0.15)	0.006	0.00 (−0.06 to 0.05)	0.945	0.01 (−0.05 to 0.07)	0.808
Helpfulness of operator	0.14 (0.04 to 0.24)	0.008	0.06 (−0.03 to 0.15)	0.204	0.04 (−0.04 to 0.12)	0.345	0.12 (0.04 to 0.20)	0.003
How operator listened	0.15 (0.05 to 0.25)	0.003	0.05 (−0.04 to 0.14)	0.268	0.00 (−0.08 to 0.09)	0.954	0.07 (−0.01 to 0.15)	0.068
Health professional call back time*	0.09 (0.03 to 0.16)	0.007	0.45 (0.39 to 0.52)	<0.001	0.05 (−0.02 to 0.11)	0.140	0.13 (0.08 to 0.19)	<0.001
Very poor/poor	Reference group		Reference group		Reference group		Reference group	
Acceptable	0.16 (−0.02 to 0.34)	0.089	0.70 (0.54 to 0.86)	<0.001	0.07 (−0.09 to 0.23)	0.376	0.38 (0.24 to 0.52)	<0.001
Good	0.34 (0.15 to 0.53)	0.001	1.05 (0.87 to 1.22)	<0.001	0.18 (0.02 to 0.35)	0.030	0.51 (0.37 to 0.66)	<0.001
Excellent	0.35 (0.14, 0.56)	0.001	1.41 (1.22 to 1.60)	<0.001	0.10 (−0.08 to 0.29)	0.271	0.48 (0.31 to 0.64)	<0.001
Not applicable	0.29 (0.07 to 0.52)	0.011	0.98 (0.79 to 1.17)	<0.001	−0.04 (−0.23 to 0.15)	0.706	0.35 (0.17 to 0.53)	<0.001
Happy with treatment option								
Yes	Reference group		Reference group		Reference group		Reference group	
No	−0.21 (−0.39 to −0.02)	0.030	−0.32 (−0.49 to −0.15)	<0.001	−0.58 (−0.73 to −0.44)	<0.001	−0.70 (−0.83 to −0.56)	<0.001
Consultation satisfaction	0.05 (−0.01 to 0.12)	0.105	0.06 (0.01 to 0.12)	0.025	0.56 (0.51 to 0.61)	<0.001	0.43 (0.38 to 0.47)	<0.001

*Sensitivity analyses excluded the ‘not applicable’ category and entered this covariate as an ordinal variable to assess the global effect on each outcome, which is reported above the effects of the separate dummy variables. Models controlled for participants’ age, gender, deprivation quintile (IMD), ethnicity (white, other ethnic group) and management option received (telephone advice, treatment centre, home visit/other), as well as the type of provider contacted (NHS, commercial, social enterprise), and were clustered by provider (n=6). The GPPS items (dependent variables) were standardised so that regression coefficients are comparable across models. For all models, n=1396.

GPPS, General Practice Patient Survey; OPQ, Out-of-hours Patient Questionnaire; IMD, Index of Multiple Deprivation.

## Discussion

This study sought to establish the construct- and concurrent validity of four items from the GPPS^21^ evaluating service users’ experience of out-of-hours care through comparisons with an established, valid and reliable measure (the OPQ).^9^ ^10^ The preliminary work highlighted the need to make minor modifications to three of the four GPPS items to improve ease of comprehension by service users and response options. The modified GPPS item-set (‘entry access’, ‘timeliness of care’, ‘confidence and trust’, ‘overall experience’) formed a single construct, which summarised the two-domain structure of the OPQ moderately well. Therefore, we believe that the GPPS item-set evaluating out-of-hours care has the potential for acceptable construct validity as a scale of overall satisfaction, given minor modifications.

Our well-controlled regression models adjusted for service users’ age, gender, deprivation quintile (IMD), ethnicity, management option received and type of provider contacted (NHS, commercial, social enterprise) and accounted for clustering of participants by the provider from which they were sampled. Each of the four outcomes was strongly associated with a distinct set of related items from the OPQ, thus demonstrating their concurrent validity. Evaluations of entry access were significantly related to ratings of the length of time before service users’ calls to the provider were answered, the helpfulness of the call operator and the extent to which the operator listened, which is supported by these items loading onto the same construct in PCAs in this study and elsewhere.^9^ ^21^ Similarly, evaluations of timeliness of care were strongly associated with the time taken for the call to be answered, but were not related to ratings of the helpfulness of the call operator. Instead, timeliness was most strongly associated with the length of time taken for a health professional to call back, an association also observed in a recent study of patient satisfaction with out-of-hours care from the Netherlands.^22^

Using GPPS data, Croker and Campbell^23^ found that patients’ confidence and trust in a health professional they consulted with in an inhours primary care setting was highly influenced by interpersonal aspects of the delivered care as reported by patients, such as having been given enough time, having felt listened to, having been given explanations about tests and treatments, having treated the patient with care and concern and having taken them seriously. In this study, analogous items from the OPQ, combined into the consultation satisfaction scale, were strongly associated with service users’ ratings of confidence and trust in the out-of-hours health professional with whom they consulted. Confidence and trust was not related to items evaluating entry access. The consultation satisfaction scale incorporated an item on ratings of the length of the consultation, which has also been shown elsewhere to be a factor in confidence and trust.^24^

Respondents’ ratings of their overall experience were strongly associated with items from all three included sections of the OPQ; entry access, the result of the service users’ call and the consultation with a health professional. Patients’ evaluations of their overall experience of inhours primary care have been shown to be most related to doctor communication and the helpfulness of receptionists.^25^ We found that service users’ ratings of their overall experience (the item unmodified from the GPPS) were strongly associated with their consultation satisfaction, which included elements of doctor communication, and also the helpfulness of the call operator.

### Strengths and limitations of study

A considerable strength of this study is the large sample of service users, which enabled us to perform reliable statistical analyses using a large number of variables. When using factor analysis, the best practice is to have 5–10 participants per measure,^18^ with a higher participant-to-measure ratio yielding more reliable results; we had upwards of 64 participants per measure. Our regression models controlled for salient participant and provider characteristics, and took into account the multilevel nature of the data (service users clustered within providers).

Although the sample was large, the overall response rate was low and responders tended to be older and less deprived, and had a higher proportion of males than non-responders. However, we do not believe that any loss of representativeness of the sample on account of these factors unduly affected the analyses reported here, which were focused on determining the structure of questions on service users’ experience and the associations between these, rather than providing incidence/prevalence rates of conditions or similar outcomes that are highly affected by such issues. Our methods controlled for these factors where possible and our findings are corroborated by the existing literature, as discussed above.

We made minor modifications to either the word stems or to response categories for three of the four GPPS items after careful piloting with service users that included the use of cognitive testing (report available from authors). Furthermore, while the GPPS asks questions to respondents about making contact with a GP out-of-hours service in the past 6 months, our respondents were asked to answer questions relating to the last time they made contact with a GP out-of-hours service, having been sampled from out-of-hours providers’ databases within 2 weeks of having made the contact. While this may limit the degree to which our findings apply to the existing GPPS survey items somewhat, we believe that this piloting was essential: Early feedback from service users identified problems in interpreting the items, and changes to two items were designed to minimise missing data through blank responses (eg, missing response categories). Our recommendations for practice are, therefore, contingent on the adjustment of current GPPS items to take account of our findings.

### Policy implications and future work

Although the National Quality Requirements for GP out-of-hours services requires out-of-hours providers to routinely audit patient experiences, no specific survey tools or methods to achieve compliance are recommended. The resultant lack of consensus precludes any attempt to benchmark the patient experience data collected by providers. Within this context, both the National Audit Office and the Care Quality Commission have recently used GPPS as an alternative data source to examine differences in patient experience of GP out-of-hours care. However, an important prerequisite to using GPPS data to benchmark services is that its psychometric properties are established. We have previously reported on the reliability of GPPS out-of-hours items.^6^ The present study demonstrated that, while composed of only four evaluative items (and after minor but essential modifications identified through cognitive testing and piloting), the out-of-hours items of the GPPS survey have both construct and concurrent validity when compared with a much longer questionnaire designed for a more detailed interrogation of out-of-hours care. Our data provide evidence that GPPS has the potential to be suitable for the purposes of national benchmarking. Furthermore, these findings suggest that patients’ evaluations of out-of-hours care, from making contact with the services through to receipt of care, can be adequately summarised in as few as four items. This indicates that monitoring patient experience of out-of-hours care is feasible even in large-scale surveys assessing multiple domains, in which space restrictions are a major constraining factor.

Identifying variations in patient experience between service commissioners and/or service providers is important in order to identify areas where service quality needs to be improved. The recent national audit noted that it was currently not possible to say whether variation in patient experience scores between Clinical Commissioning Group areas reflected differences in service quality, as opposed to variation in patient socio-demographics and case mix. We have recently published work documenting the extent to which variations in scores between GP out-of-hours services can be predicted by patient socio-demographic characteristics, as opposed to provider characteristics.^26^ This work revealed that service users’ satisfaction with care was lower when the provider was commercial, as opposed to NHS or not-for-profit, and that service users’ unable to take time away from work or from minority ethnic groups had a poorer experience of care. While suitable for the purposes of benchmarking, the brevity of the GPPS items may limit the ability of services to use these data for the purposes of identifying areas of quality improvement. We also report on the qualitative work with service providers, exploring the acceptability GPPS benchmarking has and its utility as a driver for quality improvement.^27^

## Conclusions

After minor modifications were identified through careful piloting, the four evaluative out-of-hours items of the English GPPS have the potential to be valid measures of patient experience. Four items were sufficient to adequately summarise patients’ experience of out-of-hours care, from making contact with the service to receipt of care, indicating the feasibility of monitoring even in space-constrained large-scale surveys. Although the GPPS items may be suitable for benchmarking English GP out-of-hours services, further research is needed to identify whether providing benchmark data is sufficient to assist services in identifying and implementing quality improvement initiatives.

## Supplementary Material

Web supplement

Web table 1

Web table 2

Web table 3
