# Supplementary material for: Establishing the validity of English GP Patient Survey items evaluating out-of-hours care
Source: BMJ Qual Saf. 2015 Oct 21;25(11):842–50. doi: 10.1136/bmjqs-2015-004215 (PMC5136712; doi:10.1136/bmjqs-2015-004215)
Supplement: Web supplement [file bmjqs-2015-004215-s1.pdf]

The GP Out-of-Hours Service

Please answer the questions below by putting a tick in ONE box for each question unless more than one answer is allowed (these questions are clearly marked).

We will keep your answers completely confidential.

These questions are about contacting an **out-of-hours GP service** when your GP surgery or health centre is closed (for example, in the evening, at night or at the weekend).

They are **NOT** about NHS Direct, NHS walk-in centres or Accident and Emergency (A&E) or Casualty services.

Part 1: Summary questions

Q1. On the last time contact was made with the out-of-hours GP service, who contacted the service?

- ☐ I did, for myself
- ☐ I did, for another family member
- ☐ Someone else called for me

Q2. How easy was it to contact the out-of-hours GP service by telephone?

- ☐ Very easy
- ☐ Fairly easy
- ☐ Not very easy
- ☐ Not at all easy
- ☐ Don't know/didn't make contact by telephone

Q3. How do you feel about how quickly you received care from the out-of-hours GP service?

- ☐ It was quicker than expected
- ☐ It was about right
- ☐ It took too long
- ☐ Don't know/doesn't apply

Q4. Were you prescribed or advised to take any medicines by the out-of-hours GP service you contacted?

- ☐ Yes.................................please go to Q5
- ☐ No....................................please go to Q6
- ☐ Don't know/doesn't apply...please go to Q6

Q5. How easy was it to get these medicines?

- ☐ Very easy
- ☐ Fairly easy
- ☐ Not very easy
- ☐ Not at all easy

Q6. Did you have confidence and trust in the out-of-hours health professional you consulted with?

- ☐ Yes, definitely
- ☐ Yes, to some extent
- ☐ No, not at all
- ☐ Don't know/can't say

Q7. Overall, how would you describe your experience of out-of-hours GP services?

- ☐ Very good
- ☐ Good
- ☐ Neither good nor poor
- ☐ Poor
- ☐ Very poor

Please turn over

Part 2: Detailed questions about your experience

If you did not call the service yourself, please complete by discussing the responses with the person who made the initial call to the out-of-hours service.

Please answer **ALL** the questions that apply to you by ticking the box that most closely resembles your experience. There are no right or wrong answers and the service will **NOT** be able to identify your individual responses.

Please follow the instructions carefully throughout the questionnaire, especially if you are the parent, carer, or guardian rather than the patient. Throughout the questionnaire, please tick the box that is nearest to your view.

Some questions may seem similar to the summary questions we have already asked you to complete. We are interested in finding out how much detail is useful for us to find out, so please complete all questions that apply to your care.

SECTION A – Making contact with the service

Q8. Did you contact the out-of-hours service for:

- ☐ Yourself
- ☐ Your child
- ☐ Your spouse or partner
- ☐ Another relative or friend

Q9a. Did you delay calling the out-of-hours service for any reason?

- ☐ Yes
- ☐ No............................please go to Q10a

Q9b. If yes, why:

(Please tick as many as appropriate)

- ☐ You didn't think your condition was serious enough
- ☐ You didn't want to waste anyone's time
- ☐ You weren't sure whether this was the right service to deal with your problem

Q10a. Excluding any introductory message please estimate how long it took for your call to be answered:

- ☐ Less than 30 secs
- ☐ 30 to 60 secs
- ☐ More than 60 secs

Q10b. How do you rate this?

- ☐ Very poor
- ☐ Poor
- ☐ Acceptable
- ☐ Good
- ☐ Excellent

Q11. Please rate the helpfulness of the call operator:

- ☐ Very poor
- ☐ Poor
- ☐ Acceptable
- ☐ Good
- ☐ Excellent

Q12. Please rate the extent to which you felt the call operator listened to you:

- ☐ Very poor
- ☐ Poor
- ☐ Acceptable
- ☐ Good
- ☐ Excellent

Q13. Were you told how long you might have to wait before a health professional would call you back?

'Health professionals' can include doctors, nurses, paramedics etc

- ☐ Yes
- ☐ No
- ☐ Not applicable.......please go to Section B

14a. How long did it take for a health professional to call you back?

- ☐ Less than 20 mins
- ☐ 20-60 mins
- ☐ More than 1 hour

Q14b. How do you rate this?

- ☐ Very poor
- ☐ Poor
- ☐ Acceptable
- ☐ Good
- ☐ Excellent

Q15. Did you feel able to describe your health problem over the telephone?

- ☐ Definitely not
- ☐ No, not really
- ☐ Yes, to some extent
- ☐ Yes, definitely

Q16. How do you rate the way your problem was dealt with over the phone?

- ☐ Very poor
- ☐ Poor
- ☐ Acceptable
- ☐ Good
- ☐ Excellent

SECTION B - The outcome of your call to the out-of-hours service

Q17a. What was the outcome of your most recent contact with the out-of-hours service?

(Please tick all that apply)

- ☐ I had a home visit
- ☐ I went to a treatment centre
- ☐ I had telephone advice
- ☐ An ambulance was called for me
- ☐ I was told to call an ambulance

Q17b. Were you happy with the type of care you received? i.e. home visit, treatment centre or telephone advice

(Please tick ONE box only)

- ☐ Yes, I was happy
- ☐ No, I should have had a home visit
- ☐ No, I should have been seen at a treatment centre
- ☐ No, I should have been given advice on the telephone
- ☐ Other (please specify): ....

Please turn over ☞

SECTION C - The consultation with the health professional (e.g. doctor, nurse, paramedic)

Important: this is about your **most recent contact** and includes telephone advice as well as consultations at a treatment centre or home visit

Q18. Which health professional conducted the consultation?

- ☐ Doctor
- ☐ Nurse
- ☐ Paramedic
- ☐ Don't know
- ☐ Other (please specify):..

Q19a. How long was your consultation with the health professional?

- ☐ Less than 10 mins
- ☐ 10-20 mins
- ☐ More than 20 mins

Q19b. How do you rate this?

- ☐ Very poor
- ☐ Poor
- ☐ Acceptable
- ☐ Good
- ☐ Excellent

**Please rate the following:**	*Very poor*	*Poor*	*Acceptable*	*Good*	*Excellent*	*Not applicable*
**Q20.** The **thoroughness** of the consultation.....................	☐	☐	☐	☐	☐	☐
**Q21.** The accuracy of the **diagnosis**.................................	☐	☐	☐	☐	☐	☐
**Q22.**The **treatment** you were given..................................	☐	☐	☐	☐	☐	☐
**Q23.** The **advice and information** you were given...........	☐	☐	☐	☐	☐	☐
**Q24.** The **warmth** of the health professional's manner.......	☐	☐	☐	☐	☐	☐
**Q25.** The extent to which you felt **listened to**....................	☐	☐	☐	☐	☐	☐
**Q26.** The extent to which you felt things were **explained** to you..	☐	☐	☐	☐	☐	☐
**Q27.** The **respect** you were shown...................................	☐	☐	☐	☐	☐	☐

Q28. Were you prescribed or recommended any medicines during the consultation?

- ☐ Yes
- ☐ No................................please go to Q30

Q29. How easy was it to obtain any medicines that were needed?

- ☐ Very difficult
- ☐ Quite difficult
- ☐ Neither easy nor difficult
- ☐ Quite easy
- ☐ Very easy

Q30. Did you have any problems understanding the health professional because of language barriers?

- ☐ Yes
- ☐ No

Q31a. Is English your first language?

- ☐ Yes................... please go to Section D
- ☐ No

Q31b. If no, were you offered a translation service when you contacted the out-of-hours service?

- ☐ Yes, within 15 minutes of ringing
- ☐ Yes, more than 15 minutes after ringing
- ☐ No, none needed
- ☐ No, none offered

SECTION D – Home visit

*If you received a **home visit**, please answer the following questions. If not, please go to **Section E**.*

Q32. Were you told how long you would have to wait for the health professional to visit?

- ☐ Yes
- ☐ No

Q33. Did you feel you were kept informed about the timing of your home visit?

- ☐ Yes, as much as I needed
- ☐ No, I would have liked a follow-up phone call

Q34a. How long did you have to wait for your home visit?

- ☐ Less than 30 mins
- ☐ 30-59 mins
- ☐ More than 1 hour, but less than 2 hours
- ☐ More than 2 hours, but less than 6 hours
- ☐ Over 6 hours

Q34b. How do you rate this?

- ☐ Very poor
- ☐ Poor
- ☐ Acceptable
- ☐ Good
- ☐ Excellent

SECTION E – Treatment centre

*If you attended a **treatment centre (this may be at your local hospital),** please answer the following questions. If not, please go to **Section F.***

Q35. On arrival, were you told how long you would have to wait before being seen?

- ☐ Yes
- ☐ No

Q36a. How long did you have to wait before being seen by medical staff?

- ☐ Less than 20 mins
- ☐ 20-59 mins
- ☐ More than 1 hour, but less than 2 hours
- ☐ More than 2 hours, but less than 6 hours
- ☐ Over 6 hours

Please turn over ☞

Q36b. How do you rate this?

- ☐ Very poor
- ☐ Poor
- ☐ Acceptable
- ☐ Good
- ☐ Excellent

Q37. How do you rate having your care managed at a treatment centre?

- ☐ Very poor
- ☐ Poor
- ☐ Acceptable
- ☐ Good
- ☐ Excellent

Q38a. How long did it take to travel to the treatment centre?

- ☐ Less than 15 mins
- ☐ 15-29 mins
- ☐ 30-59 mins
- ☐ An hour or more

Q38b. How do you rate this?

- ☐ Very poor
- ☐ Poor
- ☐ Acceptable
- ☐ Good
- ☐ Excellent

Q39. Were any of the following a problem for you in getting to the treatment centre?

(Please tick all that apply)

- ☐ Relying on public transport
- ☐ Arranging childcare
- ☐ Worries about my personal safety
- ☐ Cost
- ☐ Being too ill or in too much pain to travel
- ☐ Access to a car

Q40. How long did it take between being asked on the phone to go to the treatment centre and being seen by medical staff there?

- ☐ Less than 1 hour
- ☐ More than 1 hour, but less than 2 hours
- ☐ More than 2 hours, but less than 6 hours
- ☐ Over 6 hours

SECTION F – In General

Q41. Please give an overall rating of the way your care was managed by the out-of-hours service:

- ☐ Very poor
- ☐ Poor
- ☐ Acceptable
- ☐ Good
- ☐ Excellent

Q42. Do you feel your case was managed with sufficient urgency?

- ☐ Definitely not
- ☐ No, I don't think so
- ☐ Yes, I think so
- ☐ Yes, definitely

Q43. In general, before calling the out-of-hours service what sort of quality of care were you expecting?

- ☐ Very poor
- ☐ Poor
- ☐ Acceptable
- ☐ Good
- ☐ Excellent

Q44. Overall….

(Please circle the appropriate number)

I had a very poor experience — I had a very good experience

0 1 2 3 4 5 6 7 8 9 10

SECTION G – Some questions about you

The following questions ***relate to the person who had the health problem*** and will help the out-of-hours service to get the best information out of the survey. We will keep your answers completely confidential.

Q45. How old are you? ________ years old

Q46. Are you: ☐ Male ☐ Female

Q47. What is your postcode? ____________

Q48. Which ethnic group do you belong to?
(Please tick ONE box)

☐ White
☐ Black or Black British
☐ Asian or Asian British
☐ Mixed background
☐ Chinese
☐ Other ethnic group
☐ If other please specify..........................

Q49. Is your accommodation:

☐ Owner/occupied/mortgaged
☐ Rented/other

Q50. Do you have any longstanding illness, disability or infirmity?

(By longstanding we mean anything that has troubled you over a period of time)

☐ Yes
☐ No

Q51. Which of these best describes what you are doing at present?

(If more than one of these applies to you, please tick the main ONE only)

☐ Full-time paid work (30 hours or more a week)
☐ Part-time paid work (under 30 hours a week)
☐ Full-time education at school, college or university
☐ Unemployed
☐ Permanently sick or disabled
☐ Fully retired from work
☐ Looking after the home
☐ Doing something else

} Please go to Q54

Q52. In general, how long does your journey take from home to work (door to door)?

☐ Up to 30 minutes
☐ 31 minutes to 1 hour
☐ More than 1 hour
☐ I live on site

Q53. If you need to see a doctor at your GP surgery or health centre during your typical working hours, can you take time away from your work to do this?

☐ Yes
☐ No

Q54. In general, would you say your health is…?

☐ Excellent
☐ Very good
☐ Good
☐ Fair
☐ Poor

Q55. Are you a deaf person who uses sign language?

☐ Yes
☐ No

Q56. Are you a parent or a legal guardian for any children aged under 16 currently living in your home?

☐ Yes
☐ No

Q57. Do you have carer responsibilities for anyone in your household with a long-standing health problem or disability?

☐ Yes
☐ No

SECTION H – Any comments?

The space below is for you to write any additional comments you may wish to make about the out-of-hours service, which will be fed back to the service provider.

THANK YOU FOR YOUR TIME

Please return this questionnaire in the reply paid envelope (no stamp is needed)

If, for any reason, you do not have a pre-paid envelope, please return the questionnaire using the freepost address below:

Primary Care Research Group
FREEPOST RRJE-SLSG-RJSY
University of Exeter Medical School
Smeall Building
St Luke's Campus
Magdalen Road
Exeter
EX1 2LU
