# Supplementary material for: Establishing the validity of English GP Patient Survey items evaluating out-of-hours care
Source: BMJ Qual Saf. 2015 Oct 21;25(11):842–50. doi: 10.1136/bmjqs-2015-004215 (PMC5136712; doi:10.1136/bmjqs-2015-004215)
Supplement: Web table 1 [file bmjqs-2015-004215-s2.pdf]

Supplementary Table 1: Characteristics of providers recruited

ID	Provider type	Area covered	GPPS OOH satisfaction score (tertile)[1]	Average calls	
				Weekday evenings	Weekends
1	NHS	Rural	62% (mid)	150	1000
2	Social enterprise	Rural	73% (high)	424	1431
3	Commercial	Urban	66% (mid/high)	206	735
4	NHS	Inner city	41% (low)	Not available	Not available
5	Commercial	Urban	49% (low)	122	900
6	Commercial	Urban and rural	62% (mid)	110	900

[1]Scores taken from Year 5, Quarter 2 (July-September 2010) of the GP Patient Survey (GPPS) out-of-hours (OOH) items. Data available at https://gp-patient.co.uk/
