# Supplementary material for: Establishing the validity of English GP Patient Survey items evaluating out-of-hours care
Source: BMJ Qual Saf. 2015 Oct 21;25(11):842–50. doi: 10.1136/bmjqs-2015-004215 (PMC5136712; doi:10.1136/bmjqs-2015-004215)
Supplement: Web table 2 [file bmjqs-2015-004215-s3.pdf]

Supplementary Table 2: Questionnaire item response distributions

Questionnaire item	N (total=1,396)	%
How easy was it to contact the out-of-hours GP service by telephone?		
Very easy	884	63.32
Fairly easy	404	28.94
Not very easy	42	3.01
Not at all easy	15	1.07
Don't know/ didn't make contact by telephone	38	2.72
Not answered	13	0.93
How do you feel about how quickly you received care from the out-of-hours GP service?		
It was quicker than expected	605	43.34
It was about right	613	43.91
It took too long	152	10.89
Don't know/doesn't apply	13	0.93
Not answered	13	0.93
Did you have confidence and trust in the out-of-hours healthcare professional you consulted with?		
yes, definitely	928	66.48
Yes, to some extent	347	24.86
No, not at all	75	5.37
Don't know/can't say	29	2.08
Not answered	17	1.22
Overall, how would you describe your experience of the out-of-hours GP service?		
Very good	772	55.3
Good	417	29.87
Neither good nor poor	91	6.52
Poor	44	3.15
Very poor	39	2.79
Not answered	33	2.36
How do you rate [how long it took your call to be answered]?		
Very poor	30	2.15
Poor	36	2.58
Acceptable	349	25.00
Good	460	32.95
Excellent	432	30.95
Not answered	89	6.38
Please rate the helpfulness of the call operator.		
Very poor	27	1.93
Poor	20	1.43
Acceptable	215	15.40
Good	554	39.68
Excellent	496	35.53
Not answered	84	6.02
Please rate the extent to which you felt the call operator listened to you.		
Very poor	18	1.29
Poor	24	1.72
Acceptable	212	15.19
Good	549	39.33
Excellent	513	36.75
Not answered	80	5.73
How do you rate [how long it took for a health professional to call you back]?		
Very poor	34	2.44

Poor	105	7.52
Acceptable	320	22.92
Good	353	25.29
Excellent	364	26.07
Not applicable	192	13.75
Not answered	28	2.01
Were you happy with the type of care you received?		
Yes	1,187	85.03
No	149	10.67
Not answered	60	4.30
How do you rate [the length of your consultation with the health professional]?		
very poor	43	3.08
Poor	54	3.87
Acceptable	302	21.63
Good	481	34.46
Excellent	452	32.38
Not answered	64	4.58
[Please rate] the thoroughness of the consultation.		
Very poor	34	2.44
Poor	50	3.58
Acceptable	213	15.26
Good	519	37.18
Excellent	525	37.61
Not applicable[1]	9	0.64
Not answered	46	3.30
[Please rate] the accuracy of the diagnosis.		
Very poor	35	2.51
Poor	66	4.73
Acceptable	202	14.47
Good	486	34.81
Excellent	461	33.02
Not applicable[1]	71	5.09
Not answered	75	5.37
[Please rate] the treatment you were given.		
Very poor	44	3.15
Poor	58	4.15
Acceptable	181	12.97
Good	424	30.37
Excellent	450	32.23
Not applicable[1]	161	11.53
Not answered	78	5.59
[Please rate] the advice and information you were given.		
Very poor	42	3.01
Poor	64	4.58
Acceptable	197	14.11
Good	498	35.67
Excellent	513	36.75
Not applicable[1]	16	1.15
Not answered	66	4.73
[Please rate] the warmth of the health professional’s manner.		
Very poor	32	2.29
Poor	53	3.8
Acceptable	173	12.39
Good	438	31.38
Excellent	647	46.35
Not applicable[1]	4	0.29

Not answered	49	3.51
[Please rate] the extent to which you felt listened to.		
Very poor	34	2.44
Poor	50	3.58
Acceptable	163	11.68
Good	473	33.88
Excellent	624	44.70
Not applicable[1]	3	0.21
Not answered	49	3.51
[Please rate] the extent to which you felt things were explained to you.		
Very poor	32	2.29
Poor	63	4.51
Acceptable	183	13.11
Good	463	33.17
Excellent	583	41.76
Not applicable[1]	17	1.22
Not answered	55	3.94
[Please rate] the respect you were shown.		
Very poor	29	2.08
Poor	23	1.65
Acceptable	147	10.53
Good	418	29.94
Excellent	724	51.86
Not applicable[1]	10	0.72
Not answered	45	3.22

[1]"Not applicable" was the response option selected by respondents on these items.
