# Supplementary material for: Establishing the validity of English GP Patient Survey items evaluating out-of-hours care
Source: BMJ Qual Saf. 2015 Oct 21;25(11):842–50. doi: 10.1136/bmjqs-2015-004215 (PMC5136712; doi:10.1136/bmjqs-2015-004215)
Supplement: Web table 3 [file bmjqs-2015-004215-s4.pdf]

Supplementary Table 3: Reliability of the "overall satisfaction with out-of-hours care" scale formed from the four modified GPPS items.

Item	N	Item-test correlation[1]	Item-rest correlation[2]	Average inter-item correlation	Alpha
Ease of access	1345	0.697	0.451	0.538	0.777
Timeliness of care	1370	0.732	0.500	0.505	0.754
Confidence and trust	1350	0.790	0.598	0.442	0.704
Overall experience	1363	0.875	0.743	0.348	0.615
**Scale**				**0.458**	**0.772**

[1]Item-test correlations refer to the correlation between that item and the scale comprised of all the items. [2]Item-rest correlations refer to the correlation between that item and the scale comprised of the rest of the items.
